# Characterisation of an efficient atrazine-degrading bacterium, *Arthrobacter* sp. ZXY-2: an attempt to lay the foundation for potential bioaugmentation applications

**DOI:** 10.1186/s13068-018-1113-0

**Published:** 2018-04-18

**Authors:** Xinyue Zhao, Li Wang, Fang Ma, Jixian Yang

**Affiliations:** State Key Laboratory of Urban Water Resource and Environment and Environment, School of Environment, Harbin Institute of Technology, Harbin, 150090 China

**Keywords:** *Arthrobacter* sp. ZXY-2, Degradation rate, Influential factors, Bioaugmentation

## Abstract

**Background:**

The isolation of atrazine-degrading microorganisms with specific characteristics is fundamental for bioaugmenting the treatment of wastewater containing atrazine. However, studies describing the specific features of such microorganisms are limited, and further investigation is needed to improve our understanding of bioaugmentation.

**Results and conclusions:**

In this study, strain *Arthrobacter* sp. ZXY-2, which displayed a strong capacity to degrade atrazine, was isolated and shown to be a potential candidate for bioaugmentation. The factors associated with the biodegrading capacity of strain ZXY-2 were investigated, and how these factors likely govern the metabolic characteristics that control bioaugmentation functionality was determined. The growth pattern of *Arthrobacter* sp. ZXY-2 followed the Haldane–Andrews model with an inhibition constant (*K*_i_) of 52.76 mg L^−1^, indicating the possible augmentation of wastewater treatment with relatively high atrazine concentrations (> 50 ppm). Real-time quantitative PCR (RT-qPCR) results showed a positive correlation between the atrazine degradation rate and the expression levels of three functional genes (*trzN*, *atzB*, and *atzC*), which helped elucidate the role of strain ZXY-2 in bioaugmentation. In addition, multiple copies of the *atzB* gene were putatively identified, explaining the higher expression levels of this gene than those of the other functional genes. Multiple copies of the *atzB* gene may represent a compensatory mechanism that ensures the biodegradation of atrazine, a feature that should be exploited in future bioaugmentation applications.

**Electronic supplementary material:**

The online version of this article (10.1186/s13068-018-1113-0) contains supplementary material, which is available to authorised users.

## Background

Increases in the production and application of agricultural and industrial chemicals have caused rapid increases in contaminated soils and waters. Bioaugmentation is considered a suitable alternative decontamination approach to degrade organic pollutants and further accelerate the biodegradation of pollutants from wastewater [1]. The process is achieved by treating polluted waste sites with specific functional microorganisms to enhance their microbial activities [2, 3]. Bioaugmentation techniques have been commercially implemented at contaminated sites since the 1970s due to their relatively low cost and minimal impact on the environment [4–6]. However, a broad and in-depth understanding of the microbial characteristics responsible for bioaugmentation is lacking.

Atrazine, an important herbicide, is a persistent organic contaminant that has been used extensively to control both broadleaf and grassy weeds in agriculture [5, 7, 8]. Studies have shown that atrazine is highly toxic in aquatic ecosystems, and as a result, it has been classified as a toxicological contaminant, resulting in increasing concern [9]. The wastewater discharged from atrazine-manufacturing plants without treatment has led to the significant contamination of ground and surface waters [10]. Many microorganisms, primarily belonging to the genera *Pseudomonas*, *Acinetobacter*, *Rhodococcus*, *Arthrobacter*, *Bacillus*, *Variovorax*, and *Citricoccus*, have been isolated and utilised to degrade atrazine [11–18]. These strains have several common characteristics, such as high atrazine-degrading efficiency within broad pH or temperature ranges [15, 16, 19]. These characteristics suggest that the strains show promise for the bioaugmentation of atrazine-contaminated sites. However, in practice, the implementation of bioaugmentation is a complicated process that requires much effort to achieve the expected results. Understanding the successes or failures of implementation is of great importance and is necessary for future applications, requiring knowledge of the strain characteristics and mechanisms involved in atrazine degradation as a foundation. Fundamental knowledge of the process encompasses kinetic studies, pathways, gene expression, and characterisation of the whole genome. More importantly, it is critical to analyse and determine how this knowledge is related to potential bioaugmentation applications to improve the process. To date, few studies have investigated the factors determining specific characteristics of bioaugmentation strains and how these characteristics relate to the development, operation, and maintenance of bioaugmentation systems. Without fundamental knowledge and a better understanding of the bioaugmentation process, it is difficult to broadly and deeply design and implement atrazine bioaugmentation in practice.

Notably, *Arthrobacter* sp. strain ZXY-2 was reported to have a high atrazine degradation rate [17]. In this study, strain ZXY-2 was selected to investigate the factors that are important in determining a high atrazine degradation rate. To gain a better understanding of the process for future bioaugmentation applications using strain ZXY-2, our laboratory carried out studies investigating its growth kinetics and elucidated the atrazine degradation pathway in strain ZXY-2. In addition, we utilised real-time quantitative PCR (RT-qPCR) analysis to measure the transcriptional expression of three atrazine-degrading genes throughout the entire degradation process. Finally, the complete genome of strain ZXY-2 was sequenced to obtain an in-depth understanding of efficient atrazine degradation.

## Results and discussion

### Evaluation of atrazine biodegradation

Atrazine degradation by *Arthrobacter* sp. strain ZXY-2 was studied in a liquid enrichment medium. An atrazine degradation rate of 12.73 mg L^−1^ h^−1^ was obtained under the following conditions: temperature of 34 °C, pH of 9.0, inoculum size of 10% (*v/v*), sucrose concentration of 2.212 g L^−1^, and Na_2_HPO_4_·12 H_2_O concentration of 6 g L^−1^. The atrazine degradation rate of strain ZXY-2 was also examined under the following different culture conditions: an initial atrazine concentration range of 50–100 mg L^−1^, a pH range of 7.0–9.0, a carbon source range of 2–3 g L^−1^, and a temperature range of 30–35 °C. The average degradation rates of the atrazine-utilising strain ZXY-2 are shown in Additional file 1: Table S1, ranging from 7.14 mg L^−1^ h^−1^ to 12.73 mg L^−1^ h^−1^. To determine the potential atrazine-degrading capacity of strain ZXY-2, it is necessary to compare this strain with other atrazine-degrading strains that have been considered potential tools for field applications (e.g. bioaugmentation) under the same or similar culture conditions. A number of atrazine-degrading strains have been isolated and reported, and those that have been recognised as potential tools for field applications are listed in Additional file 1: Table S1. Although atrazine degradation rates were examined under various culture conditions for different strains, several conditions were kept constant to facilitate the comparison. Common conditions included an initial atrazine concentration of 100 mg L^−1^, a pH of approximately 7, a carbon source at a concentration of 3 g L^−1^, and a temperature range of 30 °C. Under these conditions, an average degradation rate of 7.14 mg L^−1^ h^−1^ was obtained for strain ZXY-2, which was higher than those of *Arthrobacter* sp. DAT1 (2.08 mg L^−1^ h^−1^), *Shewanella* sp. YJY4 (2.78 mg L^−1^ h^−1^), and *Ensifer* sp. CX-T (3.33 mg L^−1^ h^−1^) [19–21]. In addition, an average degradation rate of 9.52 mg L^−1^ h^−1^ by strain ZXY-2 was obtained, which was higher than that of *Pseudomonas* sp. ADP (4.17 mg L^−1^ h^−1^), with an initial atrazine concentration of 100 mg L^−1^, a pH of approximately 7, a carbon source concentration of 2 g L^−1^, and a temperature range of 30 °C [13]. Based on these comparisons, strain ZXY-2 demonstrated an atrazine-degrading capacity at least as effective as those of strains that have been identified as potential candidates for field applications. Thus, it is reasonable to conclude that ZXY-2 can be utilised in field applications (e.g. bioaugmentation).

### Characterisation of strain ZXY-2

#### Growth kinetics

**Fig. 1:**
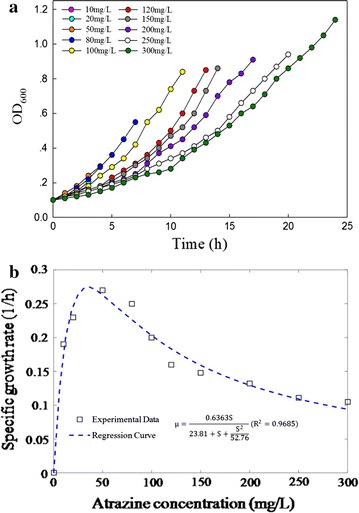
The scheme of growth kinetics with (a) time course of the growth of strain ZXY-2 and (b) fitting of the specific growth rate of *Arthrobacter* sp. ZXY-2 under different atrazine concentrations. (White square) Experimental data; (blue line) the model-fitting regression curve

The growth of strain ZXY-2 under different initial atrazine concentrations is shown in Fig. 1a. At all concentrations, ZXY-2 exhibited a corresponding increase in the OD_600_. The observed growth of strain ZXY-2 demonstrated little lag time when cultured under low atrazine concentrations (Fig. 1a), indicating that immediate degradation of atrazine was achieved. In addition, a prolonged lag phase was observed with increasing concentrations of atrazine. When the initial atrazine concentration was 50 mg L^−1^, the lag phase was only approximately 2 h, but it grew to more than 10 h at 300 mg L^−1^. This phenomenon may be attributable to the slow initiation of bacterial growth and the requirement for an acclimation period before growth accelerated at high atrazine concentrations. Atrazine degradation at different initial atrazine concentrations is shown in Additional file 1: Figure S1. At an initial concentration of 100 mg·L^−1^, little lag time was observed, and atrazine was completely removed within 12 h. However, when the initial atrazine concentration was 300 mg L^−1^, there was a lag time of 9 h, and it took 25 h for atrazine to be completely degraded. Little atrazine was degraded during the first 9 h of incubation, suggesting that it took time for strain ZXY-2 to build up resistance or adapt to the high concentration.

The prolonged lag phase indicated that high initial concentrations of atrazine may inhibit strain ZXY-2. The Haldane–Andrews model, a substrate inhibition model, was used to fit the specific growth rate ($\mu_{x}$) at different initial atrazine concentrations. The relationship between $\mu_{x}$ and the initial atrazine concentration is shown in Fig. 1b. The model fitted well with the test data (*R*^2^ = 0.9685, adjusted *R*^2^ = 0.9606, SSE = 0.001875). The kinetic parameters $\mu_{ \hbox{max} }$, $K_{\text{s}}$, and $K_{\text{i}}$ were determined to be 0.6363 h^−1^, 23.81, and 52.76 mg L^−1^, respectively. Strain ZXY-2 exhibited a high growth rate ($\mu_{ \hbox{max} }$ = 0.6363 h^−1^, see Fig. 1b) in a relatively simple culture medium. The culture medium consisted of only two components (sucrose and Na_2_HPO_4_·12 H_2_O) and was substantially more simplified than other culture media that contain more components and more complicated substances, such as vitamins and trace elements [14]. The S_m_ was determined to be 35.44 mg L^−1^. The model-fitting line showed that $\mu_{x}$ increased along with increasing atrazine concentrations from 0 to 35.44 mg L^−1^ but then decreased due to the effects of substrate inhibition. Additionally, the $K_{\text{s}}$ value was lower than the $K_{\text{i}}$ value, indicating that atrazine affinity was higher than the atrazine inhibition potential [22, 23].

#### Metabolic products and the atrazine pathway in strain ZXY-2

**Fig. 2:**
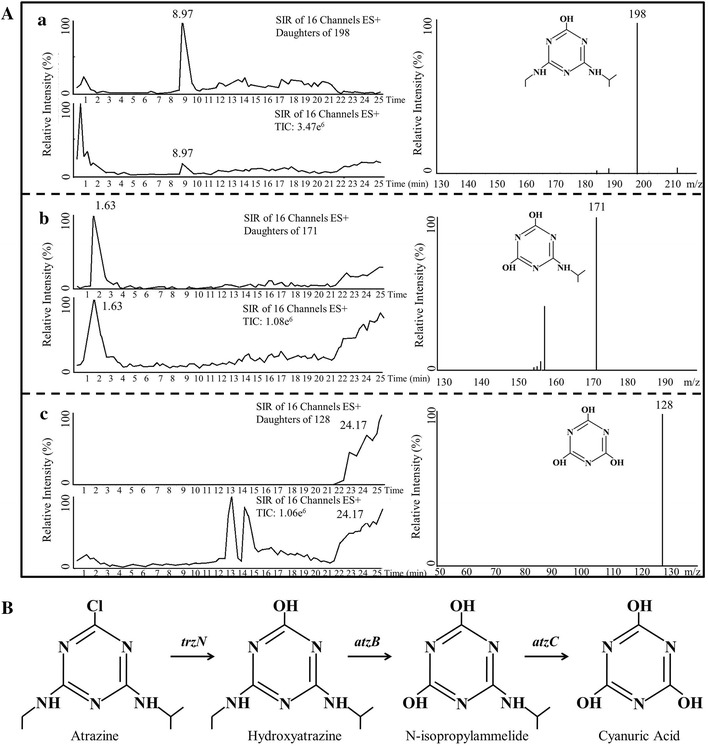
The metabolic products and pathway by strain ZXY-2. **A** HPLC-MS identification of metabolites; **B** the proposed metabolic pathway of atrazine. The analytical data for the detected ions included A (**a**) *m/z* 198, A (**b**) *m/z* 171, and A (**c**) *m/z* 128. Compounds A (**a**), A (**b**), and A (**c**) were identified as hydroxyatrazine, *N*-isopropylammelide, and cyanuric acid, respectively

**Fig. 3:**
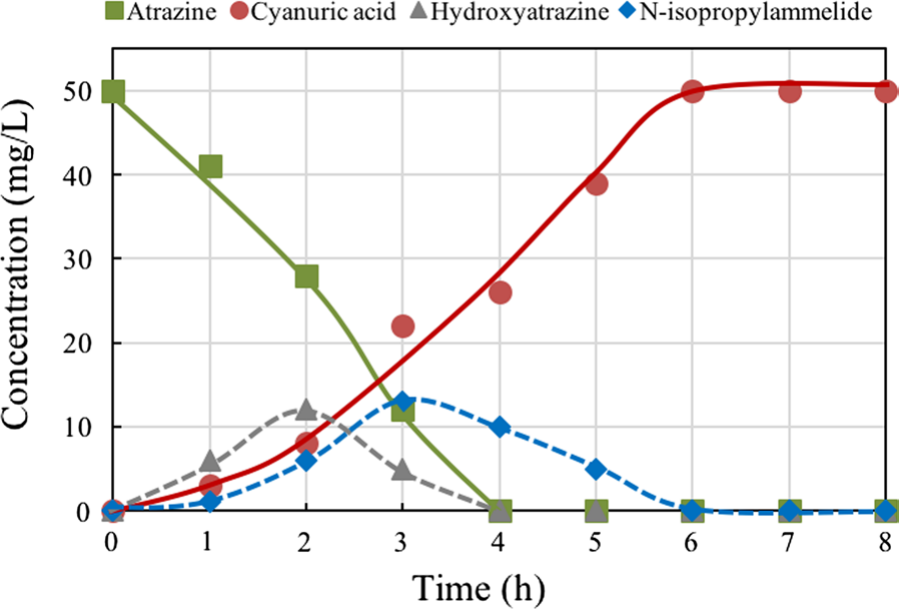
Plot of atrazine degradation by strain ZXY-2 and the evolution of its metabolites

Figure 2A shows the three main metabolites of atrazine following degradation by strain ZXY-2. The metabolites were identified as hydroxyatrazine (*m/z* of 198), *N*-isopropylammelide (*m/z* of 171), and cyanuric acid (*m/z* of 128). A putative pathway for atrazine degradation was proposed based on HPLC/MS analysis and previous studies (Fig. 2B) [24–26]. The hydrolytic pathway metabolised atrazine to hydroxyatrazine, and the N-alkyl substituents were then eliminated from the s-triazine ring to yield cyanuric acid. Figure 3 depicts the degradation of atrazine by strain ZXY-2 and the evolution of its metabolic products with time. The degradation of atrazine was 44% at 3 h, and no further degradation was observed after 4 h of incubation. Consequently, hydroxyatrazine and *N*-isopropylammelide were produced during the first few hours with different rates of production, while the concentration of cyanuric acid gradually increased with the reduction in the atrazine concentration and reached a maximum accumulation at 6 h. The results indicated that all of the atrazine was transformed to cyanuric acid. However, cyanuric acid was not further transformed into other metabolites, and thus, strain ZXY-2 was unable to completely mineralise atrazine.

#### The expression dynamics of functional genes

**Fig. 4:**
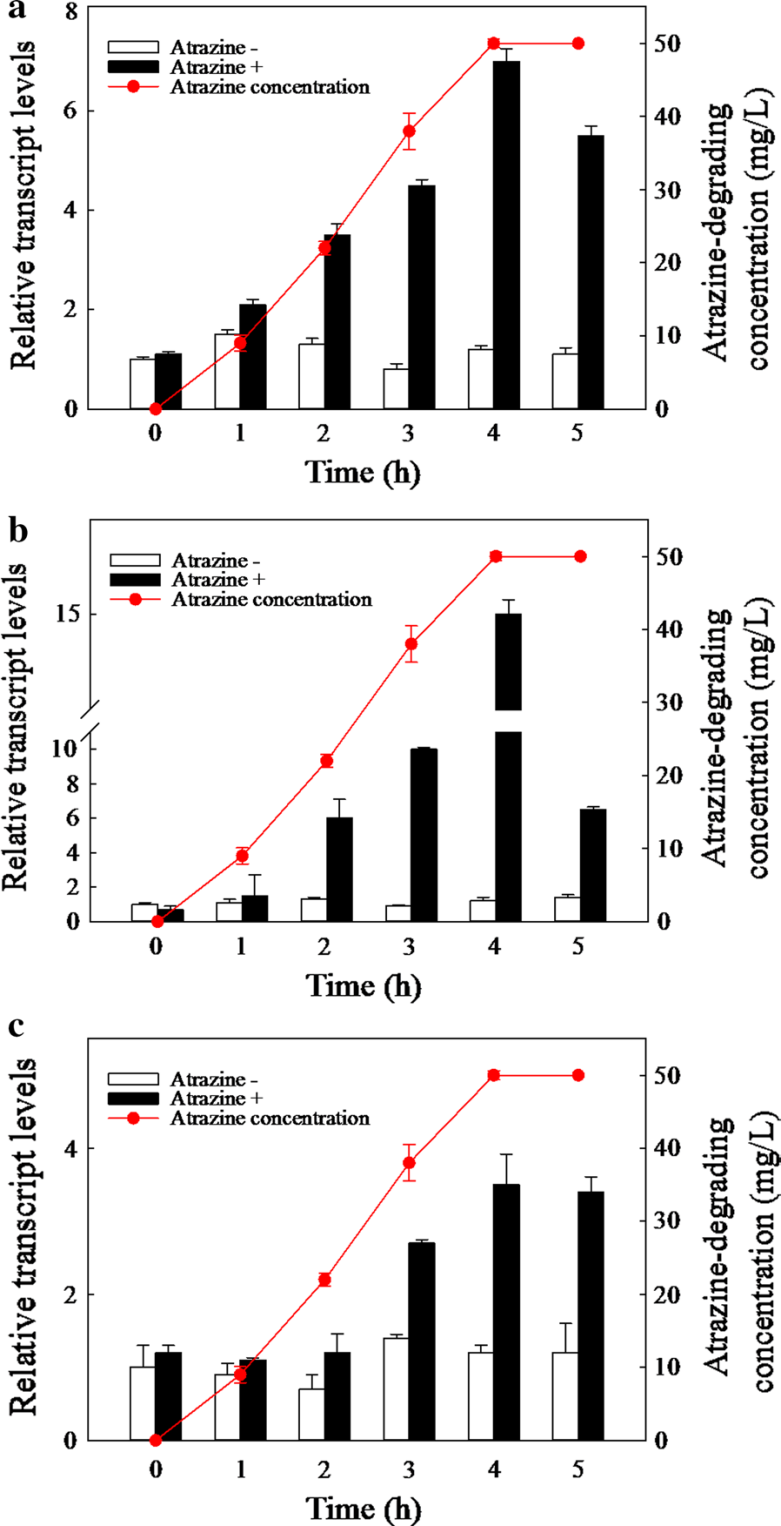
Kinetic relationships between the expression of three genes and atrazine degradation. Relative transcript levels were measured by RT-qPCR, using the 16S rRNA gene as a housekeeping gene, and are shown for (**a**) *trzN*, (**b**) *atzB*, and (**c**) *atzC*. At each kinetic sampling point, a Fisher’s test was performed to compare the relative transcript levels in atrazine-treated and control samples (*n* = 3, *p* < 0.05)

We employed RT-qPCR to more effectively quantify the differential expression of atrazine-degrading functional genes. Many atrazine-degrading genes, such as *trzN*, *atzA*, *atzB*, *atzC*, *atzD*, *atzE,* and *atzF*, have been reported. Using the primers shown in Additional file 1: Table S2, only partial genes were amplified, and the products showed size differences in agarose gels. After amplification, *trzN*, *atzB*, and *atzC* were found to have sizes of approximately 0.2, 1.4, and 0.45 kb, respectively, as shown in Additional file 1: Figure S2. No other atrazine-degrading genes, such as *atzA*, *atzD*, *atzE*, and *atzF*, were observed. Thus, only *trzN*, *atzB* and *atzC* were chosen for this analysis. According to the RT-qPCR results (Fig. 4, at 4 h), the *trzN*, *atzB*, and *atzC* genes exhibited higher expression levels than the control group (sevenfold, 13.5-fold, and 3.5-fold, respectively), suggesting the up-regulation of gene expression upon exposure to atrazine. Additionally, the differences in the expression of the three genes may be attributable to gene regulation by different promoters.

Figure 4 also illustrates the relationship between atrazine removal and the expression levels of the three functional genes. The concentration of atrazine removed closely paralleled the increased gene expression levels of all three genes, with the highest gene expression levels and the highest atrazine degradation observed at 4 h. Subsequently, decreased gene expression levels were observed after the atrazine degradation efficiency reached 100% at 5 h. The fluctuation process of expression dynamics implied that the induction effect was compromised after expression peaked, potentially due to the decrease in the concentration of atrazine along with biodegradation. Positive correlations between atrazine degradation and the gene transcript levels of *trzN*, *atzB*, and *atzC*, with R^2^ values of 0.964, 0.980, and 0.854, respectively, were established before 5 h of incubation. This result is similar to a previous finding demonstrating a positive correlation between pollutant degradation and functional gene expression [27]. The higher *R*^2^ value between *atzB* gene expression and atrazine degradation indicates that the regulation of *atzB* gene expression may be more strongly correlated with biodegradation kinetics than *trzN* or *atzC* gene regulation, suggesting an important role for *atzB* in atrazine degradation. Therefore, it can be concluded that investigating the expression levels of functional genes in strain ZXY-2 contributes to an improved understanding of the microbial degradation of atrazine.

#### Genomic characterisation of strain ZXY-2

The genome of ZXY-2 was composed of one circular chromosome and five circular plasmids with an average G + C content of 63.29%. A total of 2893 genes were classified into clusters of orthologous groups (COG) families in 22 categories (Table 1). Functional gene categories were primarily involved in energy production and conversion (7.16%), amino acid transport and metabolism (11.21%), carbohydrate transport and metabolism (11.42%), replication, recombination and repair (9.95%), inorganic ion transport and metabolism (6.12%), and transcription (8.31%). Energy metabolism-associated genes, including energy production and conversion genes, amino acid transport and metabolism genes, and carbohydrate transport and metabolism genes, with higher relative abundances than their counterparts in the complete sequences of the atrazine-degrading strains *Arthrobacter* sp. FB24 and *Arthrobacter* sp. TC1 were isolated from ZXY-2 [28, 29].

**Table 1:** Numbers of genes associated with general COG functional categories

Code	Functional category	Value	Proportion^a^
A	RNA processing and modification	1	0.04
B	Chromatin structure and dynamics	1	0.04
C	Energy production and conversion	200	7.16
D	Cell cycle control, cell division, chromosome partitioning	24	0.86
E	Amino acid transport and metabolism	313	11.21
F	Nucleotide transport and metabolism	87	3.11
G	Carbohydrate transport and metabolism	319	11.42
H	Coenzyme transport and metabolism	103	3.69
I	Lipid transport and metabolism	121	4.33
J	Translation, ribosomal structure, and biogenesis	153	5.48
K	Transcription	232	8.31
L	Replication, recombination, and repair	278	9.95
M	Cell wall/membrane/envelope biogenesis	111	3.97
N	Cell motility	18	0.64
O	Posttranslational modification, protein turnover, chaperones	101	3.62
P	Inorganic ion transport and metabolism	171	6.12
Q	Secondary metabolites biosynthesis, transport, and catabolism	86	3.08
R	General function prediction only	223	7.98
S	Function unknown	196	7.02
T	Signal transduction mechanisms	88	3.15
U	Intracellular trafficking, secretion, and vesicular transport	24	0.86
V	Defense mechanisms	43	1.54

^a^The total is based on the total number of protein-coding genes in the annotated genome

### Implications for the bioaugmentation potential of strain ZXY-2

In this study, we implemented the Haldane–Andrews model to analyse the growth kinetics of ZXY-2 and obtained an inhibition coefficient value ($k_{\text{i}}$) of 52.76 mg L^−1^. This result indicated that strain ZXY-2 grew well and metabolised efficiently without atrazine inhibition in a niche where the concentration of atrazine was below 52.76 mg L^−1^. The concentrations of atrazine in agricultural or industrial runoff are generally far below 52.76 mg L^−1^. For example, atrazine concentrations in an agricultural runoff sample were at trace levels ($\mu$g L^−1^), while those in industrial runoff were between 0.024 and 22 mg L^−1^ [30, 31]. Taken together, the $k_{\text{i}}$ value in this study provides a fundamental basis for bioaugmentation using ZXY-2 to treat atrazine-contaminated wastewater.

We expect that atrazine removal will be enhanced by inoculating strain ZXY-2 in natural settings. However, the direct degradation of atrazine by strain ZXY-2 may not be the only reason for the enhanced removal of atrazine. There may be other factors, such as horizontal gene transfer and natural genetic processes, that transfer degradation genes from inoculated strains to the indigenous microbial population [32]. To better control and maintain bioaugmentation operations, it is important to distinguish how and to what extent strain ZXY-2 performs during atrazine degradation under natural conditions. To understand the performance capability of this strain, the relationships between atrazine degradation by ZXY-2 and the expression levels of functional genes should be established. This study identified a positive correlation between atrazine degradation and functional gene expression under experimental conditions. In the future, the correlation under natural conditions must be determined. Comparing the correlations established under different conditions will help to clearly identify the role that strain ZXY-2 may play in bioaugmentation.

In this study, the nucleotide sequences of the whole chromosome genome and five plasmids of *Arthrobacter* sp. ZXY-2 were submitted to GenBank under the Accession Numbers CP017421–CP017426. The whole genome contained genes involved in the biodegradation of atrazine to cyanuric acid (*trzN*, *atzB*, and *atzC*). All three genes were located on the chromosome only, not on the *Arthrobacter* sp. ZXY-2 plasmid. In addition, as shown in Additional file 1: Table S3, the chromosome genome contained three *atzB* proteins at nucleotide positions 337934-339274 (ORF00323, Locus_tag 329), 4207292-4208764 (ORF03987, Locus_tag 4058), and 4226226-4227596 (ORF04002, Locus_tag 4073). *atzB* gene redundancy may provide a competitive advantage over a single-copy gene because a single-copy gene may be more readily lost under growth conditions that lack adequate selection pressures, as shown for the atrazine-degrading microorganism *Pseudomonas* sp. ADP [13, 33]. Redundancy is important because the loss of one copy of the *atzB* gene due to any fluctuations in quality or any abnormal conditions in wastewater may be mitigated by the presence of multiple copies, representing a compensatory mechanism to ensure the continued ability of strain ZXY-2 to convert hydroxyatrazine to *N*-isopropylammelide, a critical intermediate step required for the effective bioaugmentation of atrazine degradation.

## Methods

### Strain and culture conditions

*Arthrobacter* sp. strain ZXY-2 (*Actinobacteria*: *Micrococcaceae*) was previously isolated from the Jilin Pesticide Plant, China [17]. Strain ZXY-2 was cultured in an enrichment medium that consisted of 6 g L^−1^ Na_2_HPO_4_·12 H_2_O, 50 mg L^−1^ atrazine, and 2.212 g L^−1^ sucrose. During cultivation, the pH, temperature, shaking speed, and inoculum size were maintained at 9.0, 34 °C, 150 r min^−1^, and 10% (*v/v*), respectively. Additionally, strain ZXY-2 was subjected to different culture conditions, with an initial atrazine concentration range of 50–100 mg L^−1^, a pH range of 7.0–9.0, a sucrose concentration range of 2–3 g L^−1^, and a temperature range of 30–35 °C. The inocula for subsequent batch tests came from a microbial suspension with an OD_600 nm_ of 1.0, and the cell density was 10^8^ CFU mL^−1^. The 16S rRNA sequence of strain ZXY-2 was previously submitted to GenBank under the Accession Number KT778822 and was stored at the China General Microbiological Culture Collection Center (CGMCC) under accession number CGMCC No. 10937 [17].

### Cell growth and atrazine degradation

Cell growth and atrazine degradation of strain ZXY-2 were investigated at initial atrazine concentrations of 10, 20, 50, 80, 100, 120, 150, 200, 250, and 300 mg L^−1^, respectively. The specific growth rates ($\mu_{x}$) of strain ZXY-2 based on OD_600_ values were analysed in the cell exponential growth phase with Eq. 1, and the data were regressed using the Haldane–Andrews model (Eq. 2) for cell growth:

1 $$\mu_{x} = \frac{1}{X}\frac{{{\text{d}}X}}{\text{dt}}$$

2 $$\mu_{x} = \frac{{\mu_\text{{max}} S}}{{S + K_{\text{s}} + \frac{{S^{2} }}{{K_{i} }}}},$$

where $\mu_{x}$ is the specific growth rate (h^−1^), *X* is the cell concentration (mg L^−1^), $\mu_{ \hbox{max} }$ is the maximum specific growth rate (h^−1^), $S$ is the substrate concentration, $K_{\text{s}}$ is the substrate half-saturation coefficient (mg L^−1^), and $K_{\text{i}}$ is the substrate inhibition coefficient (mg L^−1^). In addition, $S_{\text{m}}$ is the square root of $K_{\text{i}} *K_{\text{s}}$, representing a critical substrate inhibitor concentration that defines a turning point in the degradation rate. The kinetic parameters were estimated using non-linear regression with the matrix laboratory (MATLAB, R2015b) software package.

### Identification of atrazine and its metabolites

Samples for atrazine analysis were extracted twice with an equal volume of dichloromethane, followed by a 10-min centrifugation at 10,000×*g* to remove impurities. The supernatant was filtered through a 0.22-μm disposable sterile filter (Sartorius Stedim, Germany). The atrazine concentration was quantitatively determined using HPLC, Shimadzu, Japan) equipped with a C18 column (length: 25 cm, internal diameter: 4.6 mm, Varian) at a flow rate of 1.0 mL min^−1^ (acetonitrile/water = 6/4, v/v) with an injection volume of 20 μL. The variable-wavelength UV detector was set to 220 nm.

Mass spectrometry was used to determine the masses of the metabolites. A list of putative mass-to-charge ratios (*m/z*) of the metabolic products was generated from non-target screening. Selected ion recording (SIR) mode was subsequently applied to provide a total chromatographic profile for the putative biodegradation products. The mobile phase was a mixture of acetonitrile (A) and water (B) at a flow rate of 0.4 mL min^−1^ with a linear gradient flow from 10% A/90% B to 70% A/30% B within 24 min, returning to initial conditions within an additional 12 min.

### Total RNA extraction and reverse transcription

Culture suspensions were incubated in growth media and supplemented with either 0 or 50 mg L^−1^ atrazine. Bacterial cells were harvested every hour and centrifuged at 10,000×*g* for 10 min, and the supernatants were removed. Total RNA was extracted using an RNeasy kit following the manufacturer’s instructions (Qiagen, Germany). Trace DNA was removed by treating the RNA with RNase-free RQI DNase (Promega, USA) for 1 h at 37 °C. Extracted RNA was quantified with a spectrophotometer at 260 nm (NanoDrop-1000, USA). Total RNA was reverse-transcribed into cDNA using the PrimeScript^®^ RT Reagent Kit (TaKaRa). cDNA samples were stored at − 20 °C.

### RT-qPCR analysis

Expression of the three genes identified as having a role in the atrazine degradation process was quantified in strain ZXY-2 grown with or without atrazine. The *trzN* gene is a chlorohydrolase that replaces C–Cl with C–OH to produce hydroxyatrazine, the *atzB* gene catalyses the hydrolytic transformation of hydroxyatrazine to *N*-isopropylammelide, and the *atzC* gene catalyses the hydrolysis of N-isopropylammelide to cyanuric acid. The primers used for amplification were 5′- CTG GTA GTC CAC GCC GTA AA- 3′ (forward) and 5′- CGA ATT AAA CCA CAT GCT CCA C- 3′ (reverse) for *trzN*, 5′- TCA CCG GGG ATG TCG CGG GC- 3′ (forward) and 5′- CTC TCC CGC ATG GCA TCG GG- 3′ (reverse) for *atzB*, and 5′- GCT CAC ATG CAG GTA CTC CA- 3′ (forward) and 5′- GTA CCA TAT CAC CGT TTG CCA- 3′ (reverse) for *atzC*. The 16S rRNA gene was also amplified with primers 5′- CTG GTA GTC CAC GCC GTA AA- 3′ (forward) and 5′- GAA TTA AAC CAC ATG CTC CAC- 3′ (reverse).

RT-qPCR reactions were performed using a 7500 RT-qPCR system (Applied Biosystems, USA). Reaction mixtures (20 μL) contained 10 μL of SYBR master mix, 0.2 μL of each forward and reverse primers, and 2 μL of cDNA template. Amplification reactions consisted of one cycle at 95 °C for 6 min, 40 cycles of 15 s at 95 °C, and one cycle of 60 s at 60 °C. To normalise the expression levels of the target genes, the 16S rRNA gene was used as a housekeeping gene because the expression levels of the 16S rRNA gene remain relatively stable. The procedure was consistent with that described in previous studies, which indicated that the 16S rRNA gene was the most suitable internal reference for bacterial RT-qPCR, although various types of internal standard genes have been reported [34]. All reactions were carried out in triplicate. Gene expression at 0 h was normalised to 1. Relative gene expression and relative transcript levels were calculated using the comparative C_T_ method, also known as the $2^{{ - \Delta \Delta {\text{C}}_{\text{T}} }}$ method [35, 36].

### Genome sequencing, assembly, and analysis

The complete sequence of *Arthrobacter* sp. ZXY-2 was obtained as previously described [17]. Additionally, automated gene prediction and annotation of the assembled contigs were carried out using Glimmer Version 3.02 and the NCBI Prokaryotic Genome Annotation Pipeline, respectively [37]. Each gene was functionally assigned to a category using the COG database [38].

### Statistical analysis

Statistical analysis was performed using the Statistics 17.0 software package for Windows. Analysis of variance (ANOVA) was used to test for statistically significant differences: *p* < 0.05 was defined as significant and *p* < 0.01 as highly significant. All data are shown as the mean ± standard deviation obtained using Student’s *t* test.

## Conclusions

An atrazine-degrading strain *Arthrobacter* sp. ZXY-2 was investigated in this study to identify factors related to its efficient biodegradation capacity. The growth kinetics results demonstrated that the strain was capable of degrading atrazine with a high specific growth rate of 0.6363 h^−1^. Strain ZXY-2 degraded atrazine via a hydrolytic pathway, and cyanuric acid was detected as the final product. The quantitative expression profiles of three functional genes (*trzN*, *atzB*, and *atzC*) were obtained during atrazine degradation. Genome characteristics suggested that multiple copies of the functional gene *atzB* and a high abundance of energy metabolism genes may contribute to efficient atrazine degradation.

In addition, this study discusses how the identified factors may contribute to future bioaugmentation applications using strain ZXY-2. The inhibition constant supports the use of strain ZXY-2 to bioaugment wastewater treatment when the atrazine concentration is below 52.76 mg L^−1^. The positive correlation between atrazine degradation and the expression levels of functional genes (*trzN*, *atzB*, and *atzC*) provides a basis to determine the potential role of strain ZXY-2 in future bioaugmentation applications. The likely existence of multiple copies of the *atzB* gene, identified via complete genome sequencing, may also represent a compensatory mechanism to ensure and facilitate atrazine biodegradation by strain ZXY-2 in future applications.

## Additional file

**Additional file 1.** **Figure S1.** Time courses of atrazine degradation of strain ZXY-2 containing different initial atrazine concentrations. **Figure S2.** Atrazine-degrading gene amplification of *Arthrobacter* sp. ZXY-2. **Table S1.** Comparison of degradation rates with reported atrazine-degrading strains. **Table S2.** Sequence of the primer pairs used in PCR study. **Table S3.** The detail information of three identified *atzB* proteins in the whole genome nucleotide sequence.
