# Supplementary material for: Characterisation of an efficient atrazine-degrading bacterium, Arthrobacter sp. ZXY-2: an attempt to lay the foundation for potential bioaugmentation applications
Source: Biotechnol Biofuels. 2018 Apr 18;11:113. doi: 10.1186/s13068-018-1113-0 (PMC5905105; doi:10.1186/s13068-018-1113-0)
Supplement: Supplementary file 1 — Additional file 1. Figure S1. Time courses of atrazine degradation of strain ZXY-2 containing different initial atrazine concentrations. Figure S2. Atrazine-degrading gene amplification of Arthrobacter sp. ZXY-2. Table S1. Comparison of degradation rates with reported atrazine-degrading strains. Table S2. Sequence of the primer pairs used in PCR study. Table S3. The detail information of three identified atzB proteins in the whole genome nucleotide sequence. [file 13068_2018_1113_MOESM1_ESM.docx]

**Additional material**

**Characterisation of an efficient atrazine-degrading bacterium, *Arthrobacter* sp. ZXY-2: an attempt to lay the foundation for potential bioaugmentation applications**

Xinyue Zhao, Li Wang, Fang Ma, Jixian Yang*

State Key Laboratory of Urban Water Resource and Environment, Harbin Institute of Technology, 150090, Harbin, China.

*Corresponding author at: State Key Laboratory of Urban Water Resource and Environment, School of Environment, Harbin Institute of Technology, Harbin 150090, China.

Email address: yangxj@hit.edu.cn

Telephone/fax number: 86-045186283008

**Contents**

**Materials and Methods: PCR analysis**

**List of Figures**

**Figure S1** Time courses of atrazine degradation of strain ZXY-2 containing different initial atrazine concentration.

**Figure S2** Atrazine-degrading gene amplification of *Arthrobacter* sp. ZXY-2.

**List of Tables**

**Table S1** Comparison of degradation rates with reported atrazine-degrading strains.

**Table S2** Sequence of the primer pairs used in PCR study.

**Table S3** The detail information of three identified *atzB* proteins in the whole genome nucleotide sequence.

**References**

**Materials and Methods: PCR analysis**

To determine whether strain *Arthrobacter* sp. ZXY-2 included known atrazine-degrading genes, polymerase chain reaction (PCR) analysis was performed. The detection of *trzN*, *atzB*, *atzC*, *atzA*, *atzD*, *atzE*, and *atzF* genes were measured with the primers and conditions previously reported [1, 2]. The total genomic DNA of strain ZXY-2 was extracted using QIAamp DNA Mini Kit (QIAGEN,Germany) according to the manufacturer’s instructions. The primer sequence, anneal temperatures and corresponding products size were shown in **Table S2**. The reaction conditions follows: denaturation for 5 min at 95℃, followed by 30 cycles of 95℃ for 1 min, anneal temperatures for 30 s, and a final extension for 10 min at 72℃.

**Figure S1** Time courses of atrazine degradation of strain ZXY-2 containing different initial atrazine concentration.


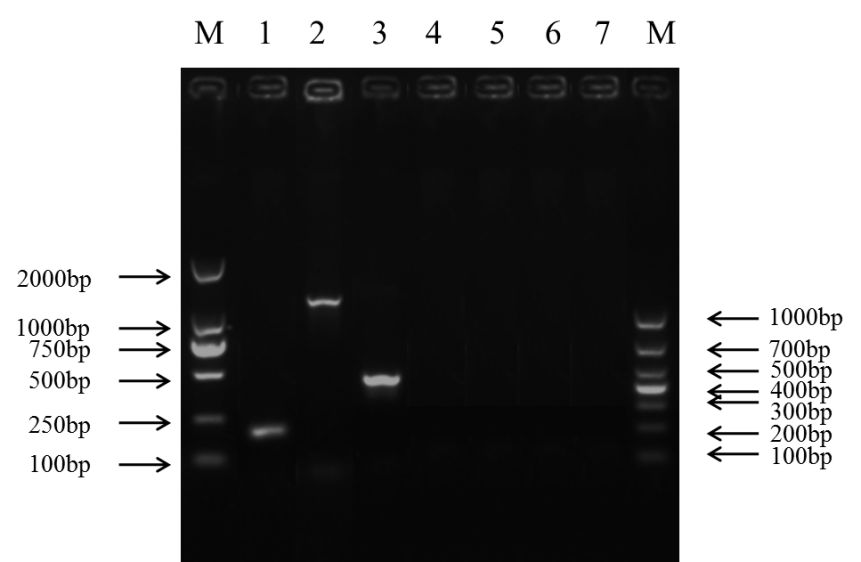


**Figure S2** Atrazine-degrading gene amplification of *Arthrobacter* sp. ZXY-2. M: DNA maker, lane 1: *trzN*, lane 2: *atzB*, lane 3: *atzC*, lane 4: *atzA*, lane 5: *atzD*, lane 6: *atzE*, lane 7: *atzF*.

**Table S1** Comparison of degradation rates with reported atrazine-degrading strains.

| Strain | Initial concentration（mg/L） | pH | Carbon source  （g/L） | Temperature  (℃) | Average degradation rate  （mg∙L^-1^∙h^-1^） | Reference |
| --- | --- | --- | --- | --- | --- | --- |
| *Pseudomonas* sp. ADP | 100 | 7.2 | 2 | 30 | 4.17 | [3] |
| *Rhodococcus* sp. N186/21 | 55 | 6.8-7.0 | 1 | 28 | 1.15 | [4] |
| *Agrobacterium radiobacter* J14a | 50 | 7.9 | 2 | 30 | 0.65 | [5] |
| *Arthrobacter* sp. AD1 | 300 | - | 3 | 30 | 6.25 | [6] |
| *Arthrobacter* sp. AD26 | 300 | 8.0 | 3 | 30 | 3.96 | [7] |
| *Arthrobacter* sp. HB-5 | 100 | 6.8 | 1 | 30 | 5.56 | [8] |
| *Arthrobacter* sp. DNS10 | 100 | - | 3 | 30 | 4.17 | [9] |
| *Arthrobacter* sp. DAT1 | 100 | 7.0 | 3 | 30 | 2.08 | [10] |
| *Bacillus subtilis* HB-6 | 200 | 7.0 | 3 | 30 | 7.50 | [11] |
| *Pseudomonas* sp. ZXY-1 | 157 | 7.14 | 3 | 31 | 19.03 | [12] |
| *Rhodococcus* sp. MB-P1 | 500 | 7.2 | - | 30 | 7.08 | [13] |
| *Arthrobacter* sp. MCM B-436 | 25 | 7.05 | 2 | 30 | 0.83 | [14] |
| *Arthrobacter* sp. AK-YN10 | 1000 | - | - | 30 | 33.3 | [15] |
| *Arthrobacter* sp. C3 | 25 | - | 1 | 30 | 0.35 | [16] |
| *Shewanella* sp.YJY4 | 100 | 7.2 | 3 | 30 | 2.78 | [17] |
| *Ensifer* sp. CX-T | 100 | 7.0 | 3 | 30 | 3.33 | [18] |
| *Citricoccus* sp.TT3 | 50 | 7.0 | 1 | 30 | 0.76 | [19] |
| *Arthrobacter* sp. ZXY-2 | 50 | 9.0 | 2.2 | 34 | 12.73 | This study |
| *Arthrobacter* sp. ZXY-2 | 50 | 7.0 | 2 | 30 | 11.10 | This study |
| *Arthrobacter* sp. ZXY-2 | 100 | 7.0 | 3 | 30 | 7.14 | This study |
| *Arthrobacter* sp. ZXY-2 | 100 | 7.0-9.0 | 3 | 20-35 | 6.94-11.25 | This study |

**Table S2** Sequence of the primer pairs used in PCR study.

| Gene | Primer | Primer sequences (from 5’ to 3’) | Annealing temperature(℃) |
| --- | --- | --- | --- |
| *trzN* | *trzN-F* | CACCAGCACCTGTACGAAGG | 55 |
|  | *trzN-R* | GATTCGAACCATTCCAAACG |  |
| *atzB* | *atzB-F* | TCACCGGGGATGTCGCGGGC | 63 |
|  | *atzB-R* | CTCTCCCGCATGGCATCGGG |  |
| *atzC* | *atzC-F* | TATTGCCCATTGTGGTGACAAC | 58 |
|  | *atzC-R* | TTGGGATTGTTGGTGACAGAAT |  |
| *atzA* | *atzA-F* | CCATGTGAACCAGATCCT | 55 |
|  | *atzA-R* | TGAAGCGTCCACATTACC |  |
| *atzD* | *atzD-F* | GGGTCTCGAGGATTTGATTG | 55 |
|  | *atzD-R* | TCCCACCTGACATCACAAAC |  |
| *atzE* | *atzD-F* | TACGCGGTAAAGAATCTGTT | 52 |
|  | *atzD-R* | GGAGACCGGCTGAGTGAGA |  |
| *atzF* | *atzD-F* | ACCAGCCCTTGAATCATCAG | 53 |
|  | *atzD-R* | ACTTACAAACGCACCGAACC |  |

**Table S3** The detail information of three identified *atzB* proteins in the whole genome nucleotide sequence.

| ORF | Locus_tag | Nucleotide position | Genome position | Qeury length | Protein description |
| --- | --- | --- | --- | --- | --- |
| ORF00323 | locus_tag 329 | 337934-339274 | chromosome | 446 | hydroxy-dechlor-atrazine |
| ORF03987 | locus_tag 4058 | 4207292-4208764 | chromosome | 490 | hydroxyatrazine hydrolase |
| ORF04002 | locus_tag 4073 | 4226226-4227596 | chromosome | 456 | triazine hydrolase |

**Reference：**

[1] M.L. de Souza, J. Seffernick, B. Martinez, M.J. Sadowsky, L.P. Wackett. The atrazine catabolism genes atzABC are widespread and highly conserved. J Bacteriol. 1998; (180): 1951-1954.

[2] M.L. de Souza, L.P. Wackett, M.J. Sadowsky. The atzABC genes encoding atrazine catabolism are located on a self-transmissible plasmid in Pseudomonas sp. strain ADP. Appl Environ Microbiol. 1998; (64): 2323-2326.

[3] R.T. Mandelbaum, D.L. Allan, L.P. Wackett. Isolation and Characterization of a Pseudomonas sp. That Mineralizes the s-Triazine Herbicide Atrazine. Appl. Environ. Microbiol. 1995; (61): 1451-1457.

[4] I. Nagy, F. Compernolle, K. Ghys, J. Vanderleyden, R. De Mot. A single cytochrome P-450 system is involved in degradation of the herbicides EPTC (S-ethyl dipropylthiocarbamate) and atrazine by Rhodococcus sp. strain NI86/21. Appl Environ Microbiol. 1995; (61): 2056-2060.

[5] J.K. Struthers, K. Jayachandran, T.B. Moorman. Biodegradation of atrazine by Agrobacterium radiobacter J14a and use of this strain in bioremediation of contaminated soil. Appl Environ Microbiol. 1998; (64): 3368-3375.

[6] B. Cai, Y. Han, B. Liu, Y. Ren, S. Jiang. Isolation and characterization of an atrazine-degrading bacterium from industrial wastewater in China. Lett Appl Microbiol. 2003; (36): 272-276.

[7] Q. Li, Y. Li, X. Zhu, B. Cai. Isolation and characterization of atrazine-degrading Arthrobacter sp. AD26 and use of this strain in bioremediation of contaminated soil. J. Environ. Sci. 2008; (20): 1226-1230.

[8] J. Wang, L. Zhu, A. Liu, T. Ma, Q. Wang, H. Xie, J. Wang, T. Jiang, R. Zhao. Isolation and characterization of an Arthrobacter sp. strain HB-5 that transforms atrazine. Environ. Geochem. Health. 2011; (33): 259-266.

[9] Y. Zhang, Z. Jiang, B. Cao, M. Hu, Z. Wang, X. Dong. Metabolic ability and gene characteristics of Arthrobacter sp. strain DNS10, the sole atrazine-degrading strain in a consortium isolated from black soil. Int. Biodeter. Biodegr. 2011; (65): 1140-1144.

[10] Q. Wang, S. Xie. Isolation and characterization of a high-efficiency soil atrazine-degrading Arthrobacter sp. strain. Int. Biodeter. Biodegr. 2012; (71): 61-65.

[11] J. Wang, L. Zhu, Q. Wang, J. Wang, H. Xie. Isolation and characterization of atrazine mineralizing Bacillus subtilis strain HB-6. PLoS One. 2014; (9): e107270.

[12] X. Zhao, L. Wang, F. Ma, S. Bai, J. Yang, S. Qi. Pseudomomonas sp. ZXY-1, a new isolated and highly efficient atrazine-degrading bacterium, and optimization of biodegradation using response surface methodology. J. Environ. Sci. 2017; (54): 152-159.

[13] Fazlurrahman, M. Batra, J. Pandey, C.R. Suri, R.K. Jain. Isolation and characterization of an atrazine-degrading Rhodococcus sp. strain MB-P1 from contaminated soil. Lett. Appl. Microbiol. 2009; (49): 721-729.

[14] P.A. Vaishampayan, P.P. Kanekar, P.K. Dhakephalkar. Isolation and characterization of Arthrobacter sp strain MCM B-436, an atrazine-degrading bacterium, from rhizospheric soil. International Biodeterioration & Biodegradation. 2007; (60): 273-278.

[15] S. Sagarkar, P. Bhardwaj, V. Storck, M. Devers-Lamrani, F. Martin-Laurent, A. Kapley. s-triazine degrading bacterial isolate Arthrobacter sp. AK-YN10, a candidate for bioaugmentation of atrazine contaminated soil. Appl Microbiol Biotechnol. 2016; (100): 903-913.

[16] H. Wang, Y. Liu, J. Li, M. Lin, X. Hu. Biodegradation of atrazine by Arthrobacter sp C3, isolated from the herbicide-contaminated corn field. Int J Environ Sci Te. 2016; (13): 257-262.

[17] J.Y. Ye, J.B. Zhang, J.G. Gao, H.T. Li, D. Liang, R.M. Liu. Isolation and characterization of atrazine-degrading strain Shewanella sp. YJY4 from cornfield soil. Lett Appl Microbiol. 2016; (63): 45-52.

[18] L.M. Ma, S.S. Chen, J. Yuan, P.P. Yang, Y. Liu, K. Stewart. Rapid biodegradation of atrazine by Ensifer sp. strain and its degradation genes. International Biodeterioration & Biodegradation. 2017; (116): 133-140.

[19] X. Yang, H. Wei, C. Zhu, B. Geng. Biodegradation of atrazine by the novel Citricoccus sp. strain TT3. Ecotoxicol Environ Saf. 2018; (147): 144-150.
